# Evaluation of Mucociliary Clearance by Three Dimension Micro-CT-SPECT in Guinea Pig: Role of Bitter Taste Agonists

**DOI:** 10.1371/journal.pone.0164399

**Published:** 2016-10-10

**Authors:** Jose Luis Ortiz, Amparo Ortiz, Javier Milara, Miguel Armengot, Celia Sanz, Desamparados Compañ, Esteban Morcillo, Julio Cortijo

**Affiliations:** 1 Department of Pharmacology, Faculty of Medicine, University of Valencia, Valencia, Spain; 2 Jaume I University, faculty of Medicine, Castellón, Spain; 3 Department of Pharmacy, University General Hospital Consortium, Valencia, Spain; 4 CIBERES, Health Institute Carlos III, Valencia, Spain; 5 Rhinology Unit, University General Hospital Consortium, Valencia, Spain; 6 Department of Surgery, Faculty of Medicine, University of Valencia, Valencia, Spain; 7 Pathological Anatomy Department, Hospital Clínico Universitario of Valencia, Valencia, Spain; 8 Teaching and Research Unit, University General Hospital Consortium, Valencia, Spain; University of Pittsburgh, UNITED STATES

## Abstract

Different image techniques have been used to analyze mucociliary clearance (MCC) in humans, but current small animal MCC analysis using *in vivo* imaging has not been well defined. Bitter taste receptor (T2R) agonists increase ciliary beat frequency (CBF) and cause bronchodilation but their effects *in vivo* are not well understood. This work analyzes *in vivo* nasal and bronchial MCC in guinea pig animals using three dimension (3D) micro-CT-SPECT images and evaluates the effect of T2R agonists. Intranasal macroaggreggates of albumin-Technetium 99 metastable (MAA-Tc99^m^) and lung nebulized Tc99^m^ albumin nanocolloids were used to analyze the effect of T2R agonists on nasal and bronchial MCC respectively, using 3D micro-CT-SPECT in guinea pig. MAA-Tc99^m^ showed a nasal mucociliary transport rate of 0.36 mm/min that was increased in presence of T2R agonist to 0.66 mm/min. Tc99^m^ albumin nanocolloids were homogeneously distributed in the lung of guinea pig and cleared with time-dependence through the bronchi and trachea of guinea pig. T2R agonist increased bronchial MCC of Tc99^m^ albumin nanocolloids. T2R agonists increased CBF in human nasal ciliated cells *in vitro* and induced bronchodilation in human bronchi *ex vivo*. In summary, T2R agonists increase MCC *in vivo* as assessed by 3D micro-CT-SPECT analysis.

## Introduction

The airways are constantly challenged by inhaled microbial pathogens. Mucociliary clearance (MCC) is the primary physical defense against inhaled pathogens, toxins and particulates in the respiratory system. Appropriate mucus production and coordinated ciliary activity are the premises for effective clearance.

There are many reasons why MCC could be impaired. The movements of the cilia can be hindered directly as occurs in cilia genetic disorders (primary ciliary dyskinesia) or by temporary dysfunction caused by airway infection or environmental influences [1–3]. The mucus layer can constitute the main problem when dehydration of the mucus leads to increased viscosity whereby the ciliary clearance becomes ineffective [4, 5]. In chronic airway diseases, hypersecretion of mucin leads to excessive amounts of mucus with an increased viscosity that is hard to clear from the airways and in severe cases can end up forming mucus plugs whereby infection or localized atelectasis can be observed [5, 6].

Eventually, the inflammation generated by defects in MCC can lead to bronchiectasis characterized by permanent dilation of the airway and thickening of the bronchial wall [7]. All severe chronic diseases involving defective MCC result in substantial morbidity in terms of dyspnoea, recurring sinopulmonary infections, and frequent and productive coughs. The close relationship with infection is also evident in patients with acute infectious exacerbation of chronic obstructive pulmonary disease where large amounts of viscous sputum are produced [5]. In this regard, many new therapies directed to improve MCC are currently under research, including those directed to increase ciliary beat frequency (CBF), to reduce mucus secretion or mucus viscosity, with the final objective to improve morbidity and clinical symptoms of upper and lower chronic lung diseases [8].

Several technologies have been developed to monitor MCC. Thus for example, inhaled radioaerosols containing insoluble technetium 99 metastable (Tc99m) labelled colloids have been employed to monitor MCC by scintigraphy. This technique has been used for many years to evaluate human pulmonary clearance studies, reflecting the combination of MCC, cough clearance or the combination of both [9], thus assessing a possible link between mucociliary dysfunction and pathophysiology of lung diseases or pharmacological challenges to the mucociliary apparatus. However, different variations of radiocolloids (albumin/sulphur particles), particle size selection, inhalation technique, gamma camera acquisition (static/dynamic/ single-photon emission computerized tomography (SPECT)), particle deposition (nasal deposition, low airways deposition), reflects the difficulties of technique application and that the choice of ‘best’ method depends upon the specific aim of the test. Furthermore, small animal MCC analysis using radiocolloid imaging has not been well explored which may limit MCC preclinical research.

The present work analyzes different radiocolloid animal three dimension (3D) micro-computer tomography (CT)-SPECT techniques, to study nasal and bronchial MCC as a potential tool to evaluate MCC abnormalities as well as new pharmacological therapies directed to improve MCC in chronic airway disorders. To this end we characterized different bitter taste-sensing receptor (T2Rs) agonists on MCC in *in vivo* animal micro-CT-SPECT radionuclear models. T2Rs activation increase CBF [10] and bronchodilation [11] providing an optimal control to characterize *in vivo* MCC techniques. The effect T2Rs agonists on *in vitro* CBF using high speed video-microscopy and ex vivo bronchial relaxation in organ baths were also analyzed to corroborate *in vivo* analysis. Results obtained in this study provides different pre-clinical models to characterizes MCC which may be of potential value to the study of different upper and lower respiratory diseases as well as to evaluate new therapies directed to improve MCC.

## Methods

### Animal experiments

Experimentation and handling were performance in accordance with the guidelines of the Committee of Animal Ethics and Well-being of the University of Valencia (Valencia, Spain). Animal studies used pathogen-free male Guinea pig (Harlan Iberica^®^, Barcelona, Spain) at 12 weeks of age. Guinea pig were housed with free access to water and food under standard conditions: relative humidity 55 ± 10%; temperature 22 ± 3°C; 15 air cycles/ per hour; 12/12 h Light/Dark cycle.

#### Nasal mucociliary transport rate (NMTR) measurement in guinea pig

Guinea pig animals (~350 g) were anesthesized with intraperitoneal mixture of ketamin (70 mg/Kg) and medetomidin (0.25 mg/kg). After a period of 10 min, the animals were placed into double-chamber plethysmograph (DCP) with conical restrainer nouse-only system adapted to guinea pig animals (250-400g animal; emka technologies, Paris, France). DCP was connected to a ventilation pump (model 683; Harvard Apparatus) to renewal of air inside the head chamber. A vacuum pump N0022AN.9E (KNF Neuberger, Freiburg, Germany) was connected to the chamber to drawn out the air introduced by ventilator. Air from ventilator and vacuum pump were filtered through a 0.1μm PESU membrane filter (Sartorius stedium biotech, goettingen, Germany). The head unit of the DCP was connected to the nebulizer head unit (Aeroneb^®^ Lab Nebulizer, Aerogen Inc. Galway, Ireland) provided with a filler cap. Nebulizer head unit was controlled with a nebulization-controller device (emka technologies, Paris, France). A final volume of 300μl with vehicle (phosphate buffer), T2Rs agonist chloroquine, denatonium or saccharine at 1 mM were deposited in the nebulizer head unit and nebulized to the nouse-only system of the chamber head unit during 15 min. The flow rate of nebulization was controlled with the nebulization-controller device to provide a 15 min cycle of nebulization. The nebulization head emits a fine-droplet (mass median aerodynamic diameter < 3μm to 0.55 mm) aerosol at low velocity without heat and propellants and without pressure or volume fluctuations. No residual liquid remained in the head unit indicating a high efficiency. Considering that the trachea of guinea pigs with mean body weight of 400 g is a 3.51 ± 0.88 cm long and 0.21 ± 0.05 cm in diameter (mean ± SEM) and that the bronchioles, alveolar ducts, and alveolar sacs are about the same diameter (0.01 to 0.015 cm), and the alveoli are approximately 0.008 cm in diameter [12]) we can assume that nebulized solution reached the whole lung volume of guinea pig. So deposition site of the drugs was nose, tracheal, bronchial and alveolar units. In this setting, diameter of <3μm to 0.55 combine impaction and diffusion processes [13]. Drug solutions were dissolved in phosphate buffer solution adjusted to pH 7.4 since pH can affect cilia motility (acid pH decrease CBF) and bronchial contractility (acid pH increase bronchial contractility).

After vehicle/drug delivery, a suspension of albumin macroaggregated Tc99m (MAA-Tc99m) (Molipharma, Valencia Spain; 10 mCi; particle diameter 5–90μm) was intranasally instilled (one droplet of 25 μL) on the nasal meatus using a micropipette. The animals were introduced into the micro-CT-SPECT (Albira, Oncovision^™^, Valencia, Spain) in supine position in a cradle made of plexiglas, and dynamic capture of micro computer tomography (CT) and micro-SPECT images were acquired each 12 min during 60 min. The time of each capture was 1min (until 300000 counts). Head and neck images were taken as follow: planar scintigraphy in anterior (A), posterior (P) left and right lateral (LL and RL), and left and right posterior oblique (LPO and RPO) views. The images were reconstructed with filtered back projection algorithm using the Albira Suite 5.0 software (OncoVision, Valencia, Spain). The combination of acquisition and reconstruction result in a final whole image matrix of 255*255*255 mm with a pixel size of 0.25*0.25*0.25 mm. 252 image sections corresponding to the whole head and neck images (3D images) were acquired in each capture and analyzed with the PMOD^™^ software selecting a whole 3D volume of interest (VOI) after radioisotope administration (basal image). Nasal mucociliary transport rate (NMTR) was calculated measuring the distance (milimeters) reached by the front of MAA-Tc99m radioparticles signal in 1 min (mm/min). The experimental groups were control group (vehicle nebulization; n = 9) and test compound groups (chloroquine 1mM; n = 9; denatonium 1mM; n = 9, Saccharin 1mM; n = 9). Drug concentrations were selected from previous studies as effective concentrations [11].

#### Bronchial mucociliary clearance analysis using ventilated Tc99m DTPA, intratrtacheal Tc99m albumin macroaggregates or ventilated Tc99m albumin nanocolloids in guinea pig

Guinea pig animals (~350 g) were anesthesized with intraperitoneal mixture of ketamin (70 mg/Kg) and medetomidin (0.25 mg/kg). After a period of 10 min, the animals were placed into double-chamber plethysmograph and drugs were nebulized as described in the NMTR section. After drug delivery, three different techniques were assayed to evaluate bronchial mucociliary clearance. 1) The animals were nebulized as described for the dugs with a solution containing 300μl diethylene-triamine-pentaacetate 10 mCi (DTPA-Tc99m) (Molipharma, Valencia, Spain) for 15 min. After DTPA-Tc99m delivery, the animals were removed from the nebulizer chamber and allowed to breathe freely. 2) MAA-Tc99m (100 μL; 10 mCi; particle diameter 5–90μm) particle suspension was intra-tracheally administered via the endotracheal route as previously outlined [14, 15]. 3) A suspension of Tc99m albumin nanocolloids (Molipharma, Valencia Spain; 10 mCi; particle diameter ≤80nm) was nebulized during 15 min as described for drugs.

For each of the three different radionuclear preparations SPECT dynamic scans were acquired each 4 min during the first 60 min, and punctual acquisitions at 2h, 4h and 6h time points on an ALBIRA micro-SPECT system (Oncovision^®^, Valencia, Sapin) using pinhole collimators and a radius of rotation of 3.5 cm. Micro-CT images were acquired to locate radioisotope signal in animal lungs. The time of each capture was 1min (until 300000 counts). Lung images were taken as follow: planar scintigraphy in anterior (A), posterior (P) left and right lateral (LL and RL), and left and right posterior oblique (LPO and RPO) views. The images were reconstructed with filtered back projection algorithm using the Albira Suite 5.0 software (OncoVision, Valencia, Spain). The combination of adcquisition and reconstruction result in a final whole image matrix of 255*255*255 mm with a pixel size of 0.25*0.25*0.25 mm. 252 image sections corresponding to the whole lung (3D images) were acquired in each capture and analyzed with the PMOD^™^ software selecting a whole 3D VOI after radioisotope administration (basal image). During dynamic capture the animals were anesthetized. All scans were performed with gamma cameras with large field-of-view detectors, equipped with low energy general purpose collimators. The analysis of MCC was performed with the PMOD^™^ software selecting a whole 3D VOI after radioisotope administration (basal image corresponding to the 100% of radiotracer lung deposition) and repeating measures at different times applying the same VOI. Quantification was performed by analyzing the number of counts in the same VOI applied to every image (counts per pixel unit) corrected for the radioactive decay of Tc99m. Computerized tomography (CT) images were captured to visualize the anatomic location of the SEPCT images. The experimental groups are defined as control group (phosphate buffer vehicle ventilation; n = 12) and test compound group (Chloroqine 1mM; n = 12; Denatonium 1mM; n = 12, Saccharin 1mM; n = 12). Bronchial MCC was calculated in each individual as the % of the lost of radiation counts on the whole VOI at each time point during the 6h period being 100% of signal the SPECT acquisition at time 0 min.

### Human experiments

Samples of nasal epithelial cells were obtained from adult healthy subjects, free of airway infections, by curettage of middle turbinate mucosa without the use of local anaesthesia to avoid any external influence as previously outlined [16]. Lung tissue was obtained from patients who were undergoing surgery for lung carcinoma. Bronchial isolation was obtained in healthy tissue as far from the tumor lesion as previously outlined [17]. The protocol was approved by the local research and independent ethics committee of the University General Consortium Hospital of Valencia (CEIC25/2015). Informed written consent was obtained from each participant.

#### Measurement of ciliary beat frequency in human nasal epithelial cells

Nasal epithelial cells from middle turbinate mucosa were introduced into 1 ml of Dulbecco’s modified Eagle’s medium (DMEM, Cambrex) supplemented with 10% foetal bovine serum, 2 mM glutamine, penicillin (100 U/ml) and streptomycin (100 mg/ml). The nasal biopsy was partially disaggregated and dissolved in this medium. Culture dishes treated with 0.5% gelatine (Corning Incorporated Costar 3513, 12-wells) to facilitate adherence of ciliated cells were used to grow 150 μl samples of nasal epithelium biopsyas previously outlined [16]. The study was conducted between 23°C and 27°C room temperature. Measurement of CBF and functional ciliated cell analysis followed previously described protocols [16]. Briefly, cells were imaged with a digital high speed video (DHSV) imaging technique using a Nikon Eclipse TS100 microscope equipped with a 40x Nikon phase-contrast objective. Contrast enhanced video images were selected and the light intensity of the selected pixels was recorded on a frame-by-frame basis. In addition, the magnitude spectrum from a fast Fourier transformation of the variation of pixel intensity signal was analysed in an average of six different regions of (3 x 3 pixels) the cilia in individual cells in a minimum of 10 cells per experiment. CBF experiments were performed with cells submerged in Hanks' Balanced Salt Soln (HBSS) (Lonza, Cambridge, UK) at an apical volume of 100 mL. After equilibration, basal CBF was determined by 10 successive measurements obtained from the same ciliated site. Growing concentrations of T2R agonists were added every 10 minutes to generate cumulative concentration response curves between 10^−8^ M to 10^−3^ M concentration.

#### Pharmacomechanical experiments on human bronchi

Segments of bronchus were dissected free from parenchymal lung tissue and preparations cut (3–4 mm length and 2–3 mm internal diameter). Preparations were used immediately for experiments. Bronchial rings were mounted in a 5 ml organ bath chamber (Pan-Lab,USA), containing Krebs-Henseleit solution (37°C), gassed with 5% CO2 in O2 at 37°C (pH 7.4), under an initial load of 1–2 g, and the isometric tension was recorded with a transducer (Grass FT03 isometric force transducer; Grass Instruments, Quincy, MA, USA) connected to a PowerLab^®^ data acquisition system (AD Instruments, Castle Hill, New South Wales, Australia), as we reported previously [18]. Bronchial preparations were equilibrated for 60–90 min with changes in bath Krebs-Henseleit solution every 20 min before any pharmacological intervention occurred. In all experiments, the bronchi were pre-contracted with 1 μM carbachol. After the plateau was reached, increasing concentrations of T2R agonists (chloroquine, denatonium, and saccharin) were then added for constructing concentration-response curves. After the last concentration level of T2R agonists, the maximum relaxation of each segment was evaluated by the addition of 0.1 mM papaverine.

### Statistical analysis

Statistical analysis was performed using Prism 6 (GraphPad Software Inc, *San Diego*, *CA*, *USA)*. Data of MCC analysis are presented as mean±SD of % radiation counts at each time point. Statistical analysis of results is carried out by analysis of variance (ANOVA) using one-way ANOVA or two-way ANOVA followed by Bonferroni test. Significance is accepted when P<0.05.

The concentration-response curves of T2R agonists increasing CBF and relaxing human bronchi were constructed and analyzed by non-linear regression analysis and by Student’s t test, as appropriate. The logEC_50_ values, defined as the concentration of drug which induces an effect equal to 50% of its own maximal effect.

## Results

### Measure of nasal mucociliary transport rate (NMTR) using micro-CT-SPECT albumin macroagregates marked with Tc99m

**Fig 1:**
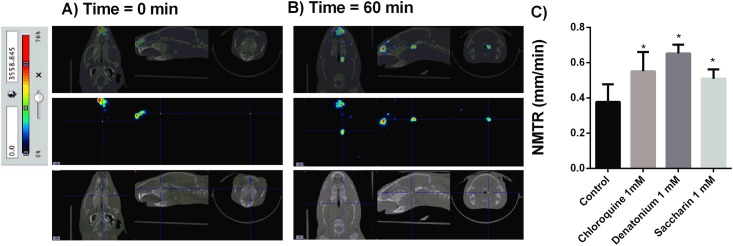
Nasal mucociliary transport rate (NMTR) measurement with combined micro-CT-SPECT macroaggregated albumin (MAA)-Technetium 99 metaestable (Tc99^m^) in guinea pig. A) MAA-Tc99^m^ traveled distance at time 0. B) MAA-Tc99^m^ traveled distance after 60 min of nasal administration. C) Bitter taste receptor agonists chloroquine, denatonium and saccharin were aerosolized during 10 min before nasal MAA-Tc99^m^ administration and the distance traveled during 60 min was monitored. Nasal mucociliary transport rate was calculated as mm/min. Representative images of n = 9 guinea pig per condition are showed. Colorimetric intensity scale adjusted for all images is shown. Results are expressed as mean ± SD. One-way repeated measures analysis of variance (ANOVA): P<0.001. Post hoc Bonferroni test: *P<0.001 compared with controls.

Anesthetized guinea pig animals were undergoing drug ventilation during 10 min with different T2Rs agonists chloroquine, denatonium or saccharine at 1 mM. After drug delivery, intranasal MAA-Tc99m (100 μL; 10 mCi; particle diameter 5–90μm) particle suspension (one droplet of 25 μL) was instilled on the beginning of nasal meatus and the distance traveled by the radioisotope signal was monitored for 60 min throughout the nasal meatus cavity. Albumin macroaggregates showed a constant velocity throughout the nasal meatus with a coefficient of variability between animal measures of 7.64 ± 0.4%. The NMTR was 0.36± 0.04 mm/min in vehicle ventilated animals (Fig 1A and 1B). The treatment with T2R agonists chloroquine, denatonium and saccharin significantly increased NMTR to 0.6±0.04 mm/min, 0.66±0.02 mm/min and 0.5±0.02 mm/min respectively (Fig 1C).

### Effects of T2R agonists on nasal CBF

**Fig 2:**
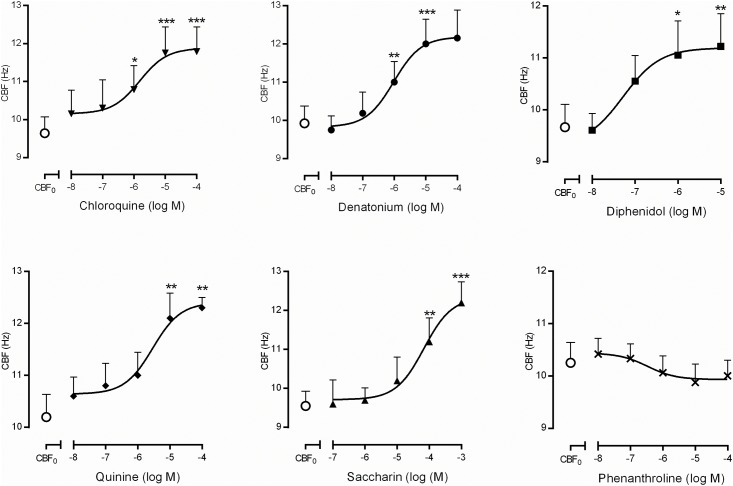
Concentration-response curves for bitter taste receptor (T2R) agonists on human nasal ciliary beat frequency CBF (Hz). CBF_0_ correspond to basal values of CBF when only vehicle was added to the culture medium. Non-selective agonists (chloroquine, denatonium, quinine, saccharin and diphenidol) induced increases in CBF at the concentrations represented, with the exception of phenanthroline. The results are expressed as a mean ± SD. Two-way repeated measures analysis of variance (ANOVA): P<0.001. Post hoc Bonferroni test: *P<0.05; **P<0.01; ***P<0.001 *vs* CBF_0_.

The basal nasal CBF of untreated cells was 9.96±0.20 Hz (n = 51). The non-selective T2R agonists induced a concentration-dependent increase of CBF with a maximum increase of 30% respect the baseline (Fig 2). The rank order of potency (EC_50_ values) was: diphenidol (0.05 μM), denatonium (0.96 μM), chloroquine (1.52 μM), quinine (2.89 μM) and saccharin (68.2 μM) (Fig 2). Diphenidol, denatonium, chloroquine and quinine at higher concentrations (>1 mM) caused ciliary incoordination perturbing CBF measurements. Phenanthroline was without effect.

### Measure of bronchial mucociliary clearance using different micro-CT-SPECT Tc99m marked radioisotopes

**Fig 3:**
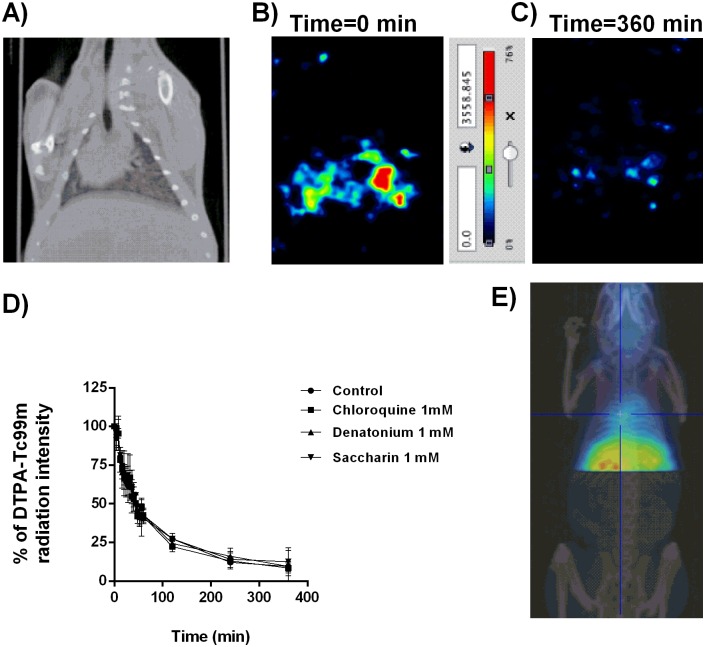
Diethylene-triamine-pentaacetate (DTPA)-Tc99^m^ evaluation as a radiotracer to monitor mucociliary clearance. A) micro-computer tomography (CT) representative image to locate SPECT images. B) SPECT representative image of nebulized DTPA-Tc99^m^ at time 0. C) SPECT representative image of nebulized DTPA-Tc99^m^ at time 360 min. D) Effects of bitter taste agonists chloroquine, denatonium and saccharin on % DTPA-Tc99^m^ radiation intensity. E) Representative image of extra-pulmonary location of DTPA-Tc99^m^ at time 360 min. Representative images of n = 12 guinea pig per experimental condition are showed. Colorimetric intensity scale adjusted for all images is shown. 100% DTPA-Tc99^m^ intensity corresponds to the start of experiments (t = 0min) for all experimental conditions). Results are expressed as mean ± SD. One-way repeated measures analysis of variance (ANOVA): P>0.05. Post hoc Bonferroni test: P>0.05 compared with controls.

Three types of Tc99m marked substances were used to evaluate bronchial MCC. First; a solution containing 10 mCi (DTPA-Tc99m) (Molipharma, Valencia, Spain) was nebulized for 15 min on a rodent nebulizer. After DTPA-Tc99m delivery, the animals were removed from the nebulizer chamber and allowed to breathe freely. Radioisotope signal was monitored throughout the lung 252 dynamic images sections obtained with the micro-CT-SPECT. Radioisotope signal showed a rapid decay because the rapid diffusion of aerosolized DTPA between the alveolar/ capillary units through the ventilation perfusion process (Fig 3A–3C). None of the T2R agonist increased bronchial MCC measures as acceleration of radioisotope signal decay (Fig 3D). SPECT images showed an irregular DTPA distribution without bronchial-tracheal expectoration throughout the measured time points, which correlates with the un-particulate nature of DTPA solution which explain the lack of activity of T2R agonists. DTPA-Tc99m distribution reached extra-pulmonary locations reflecting the passage of the radioisotope from the lung to the systemic circulation (Fig 3E) after 6h of ventilation.

**Fig 4:**
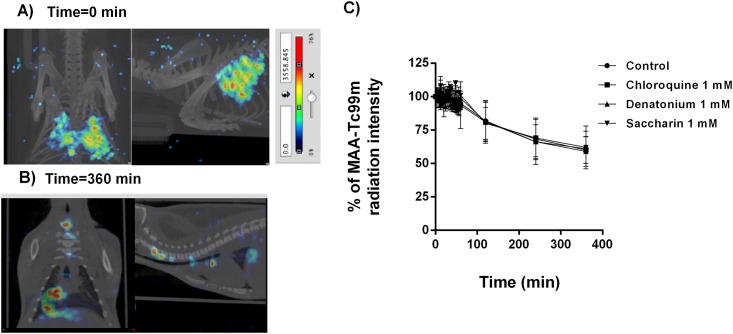
Macroaggreggate albumin (MAA)-Tc99^m^ evaluation as a radiotracer to monitor mucociliary clearance. A) Micro-CT coupled to SPECT representative image of intratracheal instillated MAA-Tc99m at time 0. B) Micro-CT coupled to SPECT representative image of intratracheal instillated MAA-Tc99^m^ at time 360 min. C) Effects of bitter taste agonists chloroquine, denatonium and saccharin on % MAA-Tc99^m^ radiation intensity. Representative images of n = 12 guinea pig per condition are showed. Colorimetric intensity scale adjusted for all images is shown. 100% MAA-Tc99m intensity corresponds to the start of experiments (t = 0min) for all experimental conditions). Results are expressed as mean ± SD. One-way repeated measures analysis of variance (ANOVA): P>0.05. Post hoc Bonferroni test: P>0.05 compared with controls.

**Fig 5:**
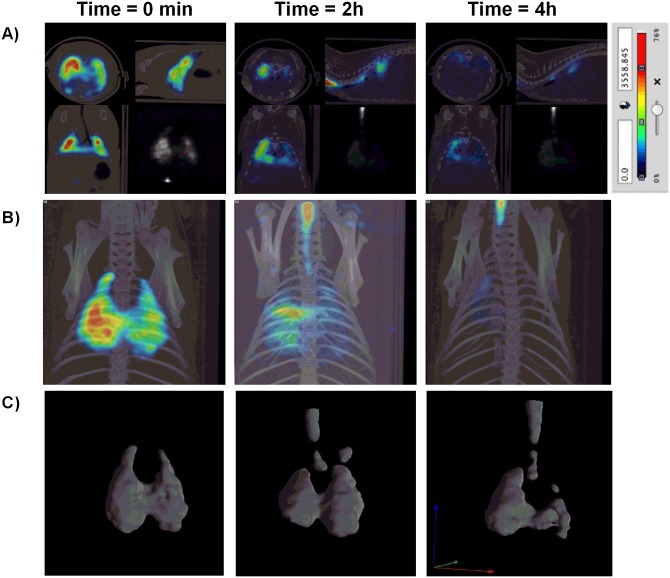
Tc99^m^ albumin nanocolloids evaluation as a radiotracer to monitor mucociliary clearance. A) Representative micro-CT-coupled SPECT two dimension sections at time 0, 2h and 4h after aerosolized Tc99m albumin nanocolloids. B) Representative micro-CT-coupled SPECT three dimension images at time 0, 2h and 4h after aerosolized Tc99m albumin nanocolloids. C) Representative SPECT three dimension images at time 0, 2h and 4h after aerosolized Tc99m albumin nanocolloids. Colorimetric Colorimetric intensity scale adjusted for all images is shown. Representative images of n = 12 guinea pig are showed.

**Fig 6:**
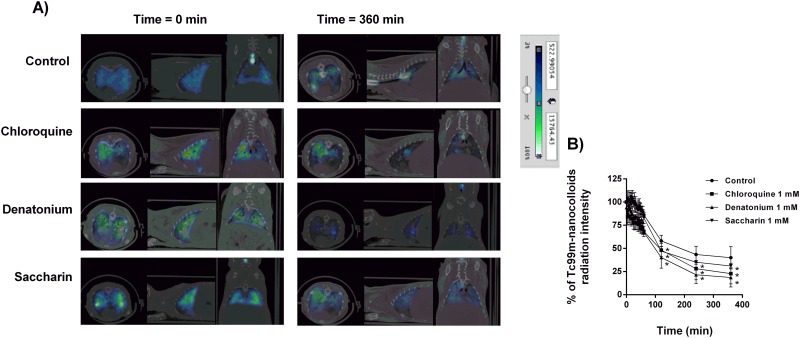
Bitter taste receptor agonists increase Tc99^m^ albumin nanocolloid bronchial mucociliary clearance in guinea pig. A) Micro-CT coupled to SPECT representative image of aerosolized Tc99^m^ albumin nanocolloids at time 0 and 360 min in presence or absence of chloroquine, denatonium and saccharin. B) Effects of bitter taste agonists chloroquine, denatonium and saccharin on % Tc99^m^ albumin nanocolloids radiation intensity. Representative images of n = 12 guinea pig per condition are showed. Colorimetric intensity scale adjusted for all images is shown. 100% Tc99m albumin nanocolloids intensity corresponds to the start of experiments (t = 0min) for all experimental conditions). Results are expressed as mean ± SD. One-way repeated measures analysis of variance (ANOVA): P<0.01. Post hoc Bonferroni test: *P<0.05 compared with controls.

The second analysis of bronchial MCC was performed using MAA-Tc99m. Intratracheal instillation of MAA-Tc99m appeared patchy and centrally deposited at the earliest time point which may reflect an elevated particle size and a lack of small bronchi distribution of albumin macroaggregates (5–90μm; Fig 4). The radioisotope signal only decayed ~ 40% of the initial signal at the end of the 6h evaluation period (Fig 4). Furthermore, none of the T2R agonists increased bronchial MCC suggesting incapacity of cilia to move albumin particle. The third analysis was carried out with a suspension of Tc99m albumin nanocolloids (particle diameter ≤80nm) that was nebulized during 15 min. Inhalation of Tc99m albumin nanocolloids showed a homogeneous distribution filling up the entire lung volume (Fig 5A). Physiological MCC was monitored by combined micro-CT and SPECT images showing continuous clearance of Tc99m albumin nanocolloids throughout the small bronchi to the tracheal section of lung reflecting the bronchial MCC in the three dimension images of the animal lungs (Fig 5B and 5C). Non-treated animals showed a rapid loss of radioisotope signal during the first 2h of inhalation followed by slow decreased signal until 6h reaching the 49.9±2% of the initial signal (Fig 6). T2R agonists accelerated the albumin nanocolloid clearance showing 28.7±1.1%, 22.5±0.24% and 38±7% of the initial signal for chloroquine, denatonium and saccharin respectively at the end of 6h period (Fig 6).

### Effects of T2R agonists on human bronchi relaxation

**Fig 7:**
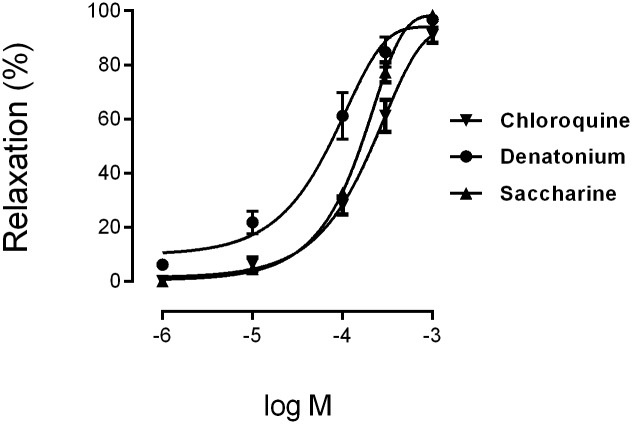
Concentration-response curves for bitter taste receptor agonists (chloroquine, denatonium and saccharin) on human bronchi. The results are expressed as a percentage of the relaxation observed with 0.1 mM papaverine (mean ± SD) of n = 4 human bronchi per condition.

Chloroquine, denatonium and sacharin produced a concentration-dependent relaxation of the carbachol-induced tone of human bronchi that achieve 90% to 100% of maximum relaxation induced by papaverine. The rank order of potency as broncodilators (EC_50_ values) was, denatonium (50 μM), saccharin (180 μM) and chloroquine (220 μM) (Fig 7, Table 1).

**Table 1:** Maximum relaxation (Emax) and potency (log EC50)observed with non-selective T2R agonists on human bronchi.

	Emax (%)	log EC_50_	n
**Chloroquine**	91.0±2.8	-3.662±0.05	8
**Denatonium**	96.8±1.6	-4.310±0.13	7
**Saccharin**	98.3±1.7	-3.741±0.44	9

The comparison of EC_50_ values relaxing human bronchi *vs* EC_50_ values increasing CBF for chloroquine (220 μM *vs* 1.52 μM) and denatonium (50 μM vs 0.96 μM) showed that potency was 100x and 50x higher stimulating ciliary motility, respectively.

## Discussion

The present work evaluates micro-CT coupled-SPECT three dimension technique to measure MCC in guinea pig animals, on both, upper and lower airways which may be of potential value as a preclinical model to evaluate compromised MCC in airway diseases as well as to evaluate the effect of drugs on *in vivo* MCC. In this regard, we observed that T2R agonists increased MCC *in vivo* by a combined mechanism including the increase of CBF of epithelial ciliated cells and bronchodilation.

Various methodologies have been used to evaluate *in vivo* lung deposition of different substances in small animals. Gamma scintigraphy [19], X-SPECT [20], positron emission tomography (PET) [21], magnetic resonance Image (MRI) [22] and fluorescence imaging [23] can measure total lung deposition and oropharyngeal deposition of particles directly by using radionuclides non-ionizable radiation and fluorescent dye. However, to our knowledge, micro-CT coupled-SPECT technique has not been used to analyze nasal or MCC in preclinical small animals. The advantage of this technique includes a better precision of data that includes different nasal or lung section analysis (252 image slices) which produces 3D images compared to the traditional 2D image producing technique, gamma scintigraphy or X-SPECT. 3D images allow more detailed data on regional lung deposition and can monitor the trachea-bronchial release of particles or the velocity of nasal mucociliary transport since it is possible to visualize the travelled length of particles. One of the best characterized radiotracer is the DTPA-Tc99m solute that has been used to monitor ventilated lung regions to calculate ventilation/perfusion ratio [24] in pulmonary embolisms, but also to study the alveolar wall integrity, nasal, tracheal and bronchial absorption and permeability [25, 26]. However the utility of DTPA-Tc99m to measure MCC is under debate. While, some authors have observed that mucociliary transport mechanism does not contribute to radiotracer clearance [25], others use DTPA-Tc99m to measure it [27]. In the present work we observed a rapid pulmonary clearance of DTPA-Tc99m that was mediated by broncho-alveolar absorption to systemic circulation rather than MCC, since hepatic radiotracer signal capture was detected at 6h post-inhalation and no movement of tracer was detected through the tracheo-bronchial apparatus. Furthermore, the stimulation of T2R did not increase physiological DTPA-Tc99m clearance which indicates that T2R activation does not affect pulmonary permeability and that DTPA-Tc99m radiotracer is not cleared by MCC transport mechanism.

Next experiments were directed to evaluate nasal and bronchial MCC using a suspension of MAA-Tc99m (particle diameter 5–90μm). Nasal administration of MAA-Tc99m showed a constant movement through nasal meatus at 0.36 mm/min reflecting a useful tool to monitor NMTR *in vivo* that has not been described previously. NMTR shows a broad variability in the literature ranging from 2.4 mm/min to 21.5 mm/min in humans [13] and 5.1 mm/min to 1.9 mm/min in rats [13]. Discrepancies observed in this work and the data reported in the literature can be explained by different techniques used, the animal species studied and also because the published work is focused in tracheal and bronchial MCC velocities but not in nasal meatus section.

In our study, the T2R agonist chloroquine and denatonium increased NMTR. Saccharin showed the lowest activity on NMTR probably because activates sweet receptors as well as T2R, so could blunt some of the bitter effects of the compound [28]. The effect of T2R agonists increasing NMTR is mediated by a direct mechanism on CBF. In fact, in isolated human nasal ciliated cells T2R activation increased CBF. Furthermore, T2R agonists can activate solitary chemosensory cells that reach the surface of the nasal epithelium and form synaptic contacts with trigeminal afferent nerve fibers [29]. Solitary chemoreceptor cells express T2R and its stimulation can induce the increase of inflammation of the nasal mucosa, the depression of respiratory rate and the increase of MCC lowering mucus viscosity in response to T2R agonists such as pseudomonas aeruginosa quorum [30–32].

In the airway, T2Rs are also expressed in bronchial smooth muscle cells of mouse [33, 34], guinea-pig [35] and human isolated airway preparations [11, 36–39] where they mediates bronchodilation. In human nose mucosa [40] have been described at least 22 types of T2Rs [41]. It has been described that these compounds augment CBF in human bronquial epithelial cells [10], but the effect on human sinonasal epithelial cells is unclear.

The T2Rs vary in their selectivity towards bitter taste compounds. Braun et al. [40] described, the following rank order expression of T2R subtypes in the human nasal respiratory mucosa: T2R5 > 4 > 3 > 31 > 10 > 14 > 13 > 46 > 43 >39 > 7. In our study, we used non-selective T2R agonists to cover the widest range of T2R with higher expression in the human nose [40]. Most of the agonists used in the present study activated T2R3, 4, 7, 10, 13, 14, 31, 39, 43, 46 and 47. Previous observations showed that the T2R agonist denatonium stimulated calcium-dependent CBF in human bronchial ciliated cells [10]. However, Lee et al. [42] reported that application of 10 mM denatonium benzoate slowed CBF in sinonasal cells suggesting that sinonasal ciliated epithelial cells do not express functional denatonium responsive T2Rs. In this work, T2R concentration higher than 1mM caused an uncoordinated CBF which was translated in a decrease of CBF, as observed by high speed video-microscopy, which may explain previous observations on decreased CBF as an over-activation of T2R.

In this study, the most potent T2R agonist increasing CBF was diphenidol (agonist of T2R4, 10, 14, 31, 39, and 40), followed by denatonium (T2R4, 10, and 39), chloroquine (T2R3, 10 and 39), quinine (T2R4, 10, 14, 31, 39, and 40) and saccharin (T2R31, 43, and 44). However, phenanthroline (selective agonist of T2R5) did not modified CBF which implicate multiple T2R subtypes, but not all, in this effect.

In contrast to nasal MCC, the use of MAA-Tc99m to measure bronchial MCC was not effective. Intratracheal instillation administered via the endotracheal route is a non-invasive aerosol pulmonary delivery technique which allows depositing particle suspensions in low airways avoiding traditional invasive surgical intratracheal administration [15]. In this work, intratracheal instillation of MAA-Tc99m appeared patchy and centrally deposited with a slow clearance that was not modified with the T2R administration, remaining ~75% of retention of MAA-Tc99m in the lung of guinea pig after 6h of instillation. As show Fig 4B, MAA-Tc99m showed a good tracheal clearance but not in bronchial tree. This phenomenon could be explained by the anatomy of guinea pig tracheo-bronchial tree which is 2100 ± 500 μm in tracheal diameter allowing a good tracheal transport. However, bronchi, bronchioles or alveolar ducts smaller diameter (100–150 μm) could retain MAA-Tc99m particles being trapped possibly by binding to the bronchial tissue.

Previous lung MCC experiments have determined that most of the particles deposited in the rat bronchial tree are cleared after about 6 to 8 h of inhalation [13]. However, other data demonstrated that intratracheal instillation particle remained with a high percentage of retention in rats when compared with nose-only aerosol inhalation [19] which may be explained by the method used for particle delivery or by the elevated particle size. In fact, the elevated particle size of the MAA-Tc99m particles, together with the incapacity of T2R to clear radiotracer, indicates the incapacity of bronchial ciliated cells to clear elevated particle size. These results are apparently contrary to that observed in nasal meatus which may be explained by the anatomical differences between the nasal cavity and bronchial tree.

In this regard, other particles suspensions joined to Tc99m have been employed to analyze MCC. Thus for example, Tc99m labeled sulfur colloid (Tc99m-SC; particle size < 100nm [43]) has been used thoroughly to evaluate MCC by means of gamma scintigraphy imaging procedure [19]. Similar results have been obtained with Tc99m albumin nanocolloids (particle diameter ≤80nm) in humans [44]. In the present work, MCC was characterized by a rapid first trachea-bronchial MCC followed by a more sustained and slow clearance of small bronchial particles [13]. Mechanism involved in lung particle clearance was attributed mostly to the MCC mechanism rather to the increase on pulmonary permeability since radiotracer particles were observed ascending from the bronchi to the trachea in a time-dependent manner. However we cannot exclude some Tc99m albumin nanocolloid pulmonary absorption to the systemic circulation. It is interesting to note that T2R agonist increased bronchial MCC at concentration that were also responsible to the increase of CBF and bronchodilation. Dual mechanism involving combination of bronchodilation and ciliary movement has been described previously for T2R agonists and could synergized increasing MCC. In chronic airway diseases such as asthma or chronic obstructive pulmonary disease (COPD), increased viscosity and mucus overproduction can end up forming mucus plugs whereby infection or localized atelectasis can be observed. Narrowing of airways combined with rubbery mucus can form mucus plugs obstructing mucociliary clearance. In this context, bronchodilation can increase the airway diameter, and with the aid of transient increases in expiratory air flow favours that mucus trapped can be released [45].

T2R agonists have been shown to induce relaxation of airway smooth muscle in animal [11, 33, 35, 39] and human [11, 33, 36, 37] at concentrations in the range observed in this work. One limitation of these compounds as bronchodilators is their low potency [37], what limits its clinical use. The results for chloroquine, denatonium and saccharin are consistent with other studies in human bronchi, showing bronchodilation with EC_50_ values in the mM range [36]. In contrast, these compounds were more potent increasing CBF, so the potency stimulating CBF for chloroquine and denatonium was 100x and 50x higher respectively. This implies that clinical effect on CBF could be achieved at lowest doses than bronchodilation. It could be difficult to understand, why EC_50_ of bitter compounds for bronchodilation and CBF differ so much?. In this regard, T2R activation increases CBF through the increase of intracellular calcium in the cilia. However T2R activation induces bronchodilation through other mechanisms such as reduction in calcium oscillation by activation of calcium-activated potassium channels (BK_Ca_) [11]. Also CBF system implicates a rapid diffusion of drugs to be directly in contact with cilia, unlike human ring bronchi that needs diffusion through the bronchial epithelium and basement membrane to be in contact with bronchial smooth muscle.

In summary, nasal administration of MAA-Tc99m particles monitored through the micro-CT coupled-SPECT is a useful technique to analyze *in vivo* NMTR, while Tc99^m^ albumin nanocolloids are preferred to the study of low airways MCC. The use of T2R agonists in this *in vivo* model corroborated previous *ex-vivo/ in vitro* findings showing an increase of NMTR and bronchial MCC.

## Supporting Information

S1 Fig Raw data from Fig 1. (XLSX)

S2 Fig Raw data from Fig 2. (XLSX)

S3 Fig Raw data from Fig 3. (XLSX)

S4 Fig Raw data from Fig 4. (XLSX)

S5 Fig Raw data from Fig 6. (XLSX)

S6 Fig Raw data from Fig 7. (XLSX)
